# Defining the remarkable structural malleability of a bacterial surface protein Rib domain implicated in infection

**DOI:** 10.1073/pnas.1911776116

**Published:** 2019-12-09

**Authors:** Fiona Whelan, Aleix Lafita, Samuel C. Griffiths, Rachael E. M. Cooper, Jean L. Whittingham, Johan P. Turkenburg, Iain W. Manfield, Alexander N. St. John, Emanuele Paci, Alex Bateman, Jennifer R. Potts

**Affiliations:** ^a^Department of Biology, The University of York, YO10 5DD York, United Kingdom;; ^b^European Molecular Biology Laboratory, European Bioinformatics Institute, CB10 1SD Hinxton, United Kingdom;; ^c^York Structural Biology Laboratory, Department of Chemistry, The University of York, YO10 5DD York, United Kingdom;; ^d^Astbury Centre for Structural Molecular Biology, The University of Leeds, LS2 9JT Leeds, United Kingdom

**Keywords:** Rib domain, domain atrophy, protein rod formation

## Abstract

*Streptococcus* groups A and B cause serious infections, including early onset sepsis and meningitis in newborns. Rib domain-containing surface proteins are found associated with invasive strains and elicit protective immunity in animal models. Yet, despite their apparent importance in infection, the structure of the Rib domain was previously unknown. Structures of single Rib domains of differing length reveal a rare case of domain atrophy through deletion of 2 core antiparallel strands, resulting in the loss of an entire sheet of the β-sandwich from an immunoglobulin-like fold. Previously, observed variation in the number of Rib domains within these bacterial cell wall-attached proteins has been suggested as a mechanism of immune evasion. Here, the structure of tandem domains, combined with molecular dynamics simulations and small angle X-ray scattering, suggests that variability in Rib domain number would result in differential projection of an N-terminal host-colonization domain from the bacterial surface. The identification of 2 further structures where the typical B-D-E immunoglobulin β-sheet is replaced with an α-helix further confirms the extensive structural malleability of the Rib domain.

## Significance

Proteins attached to the surface of group A and group B streptococcal human pathogens that contain tandemly arrayed Rib domains have been associated with invasive infections and proposed as potential vaccine candidates. Here, we present structures of the Rib domain, both isolated and in tandem. The Rib domain structure is revealed as a rare example of “domain atrophy” from the much more common immunoglobulin-like fold. Tandem Rib domains adopt a head-to-tail arrangement with limited interdomain flexibility, suggesting that the previously observed, and proposed immune evasion-related, variation in Rib domain number is likely to result in differential projection of the N-terminal host colonization domain from the bacterial surface.

Group B *Streptococcus* (GBS) *Streptococcus agalactiae* is a leading cause of sepsis and meningitis in neonates and is associated with high morbidity and mortality in early onset disease (1). Group A *Streptococcus* (GAS) *Streptococcus pyogenes* causes pharyngitis, scarlet fever, skin infections, cellulitis, necrotizing fasciitis, toxic shock syndrome, glomerulonephritis, and rheumatic fever (2) and is a common cause of severe puerperal sepsis (3).

Most invasive GBS infections result from infection with type III strains expressing a cell surface protein antigen called Rib (resistance to proteases, immunity, group B) (UniProt: P72362) (4). Rib belongs to the “alpha-like proteins of GBS” family, that also includes alpha C, and that has similar overall organization, including related N termini followed by a series of identical (or near identical) Rib domains (Pfam: PF08428) (5, 6). GAS serotype M28 expresses a surface protein R28 which is related to Rib (7) (UniProt: Q9XDB6) and promotes binding to cervical epithelial cells (7) through an N-terminal integrin-binding domain (8). An integrated analysis of diverse invasive GAS strains has identified a single nucleotide intergenic insertion resulting in significantly higher R28 transcription associated with larger lesions, increased resistance to immune clearance, and decreased survival (9). Antibodies against (4), or intranasal immunization with, Rib (10) conferred protection in animal models of GBS infection while antibodies to R28 conferred protection in an animal model of *S. pyogenes* infection (7). Cross-protective immunity has been observed on immunization with Rib and R28 against strains expressing the noncognate antigen (11). In a comparison of maternal and neonatal isoforms of the alpha C protein, the neonatal isoform was smaller, due to decreased numbers of Rib repeats (12). Rib repeats have very high DNA sequence identity, and recombination events have been proposed to facilitate domain number variability for host immune evasion and altered pathogenicity (12–14).

Despite this evidence for a role in infection and potential utility as a vaccine candidate, to date, the structure of the Rib domain has remained unknown. Here, we study Rib domains from Rib, R28, and from a surface protein from *Lactobacillus acidophilus*. Together, these 5 structures of homologous Rib domains, and further sequence analyses, show Rib is an example of domain atrophy (15), a rare event in domain structural evolution involving the loss of core secondary structure elements, and also reveal the remarkable structural malleability of this domain family. The structure, molecular dynamics simulations, and small angle X-ray scattering of a Rib domain pair suggest tandemly arrayed domains are likely to form an elongated rod on the bacterial cell surface, thus suggesting a possible mechanism of immune evasion mediated through domain number variation.

## Results and Discussion

### Rib Domain Distribution and Protein Organization.

**Fig. 1.**
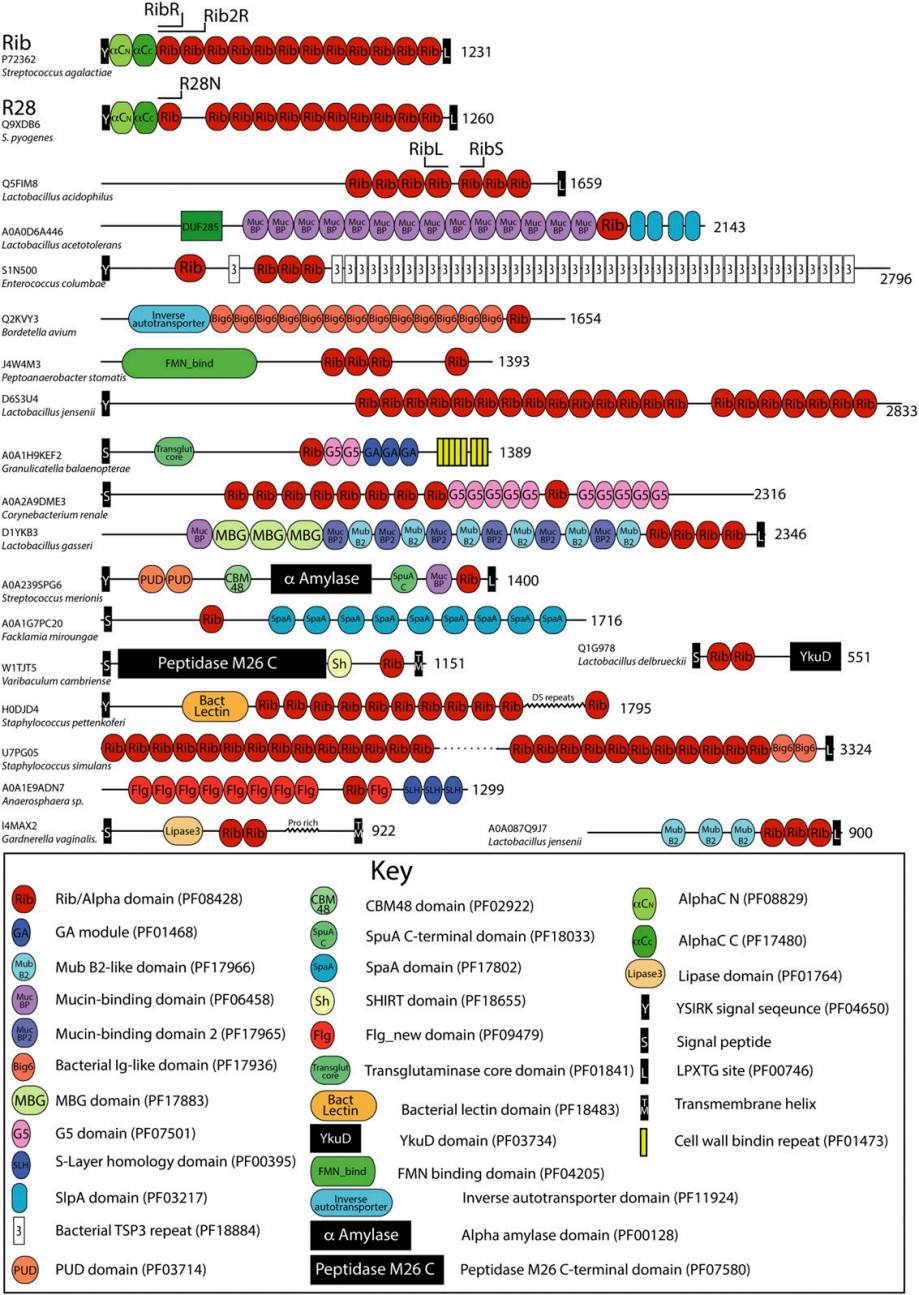
The diverse protein domain architectures of Rib domain-containing proteins. Sequence analysis of selected examples (alpha-like family, as well as other proteins) is shown illustrating the biological utility of this fold (Pfam: PF08428). Pfam domains are shown, as well as additional domains inferred from sequence searches of regions lacking domain annotation. Further domains were inferred by inspecting the domains defined by the InterPro database, as well as additional domains inferred from inspection of protein sequence repeats. The domain architecture of *S. agalactiae* Rib, *S. pyogenes* R28, and Q5FIM8 proteins are at the top of the figure, with the domains used for structure solution indicated.

The first defined Rib domain (Pfam: PF08428) features in a diverse range of bacterial proteins and functional contexts (Fig. 1) and is present in predominantly Firmicutes and Actinobacteria, with a smaller number in Proteobacteria. Rib-containing proteins frequently feature N-terminal export signals, sortase-dependent cross-linking (LPXTG) motifs (16), S-layer homology domains (17), and a wide variety of other cell surface domains, suggesting that Rib domains are surface exposed (Fig. 1).

### Structure of RibR and R28N.

Structures of single Rib domains with ∼38% sequence identity were solved: RibR (*S. agalactiae* Rib; amino acids 230 to 308) and R28N (*S. pyogenes* R28; amino acids 230 to 307) (Fig. 1 and *SI Appendix*, Fig. S1*A*).

**Fig. 2.**
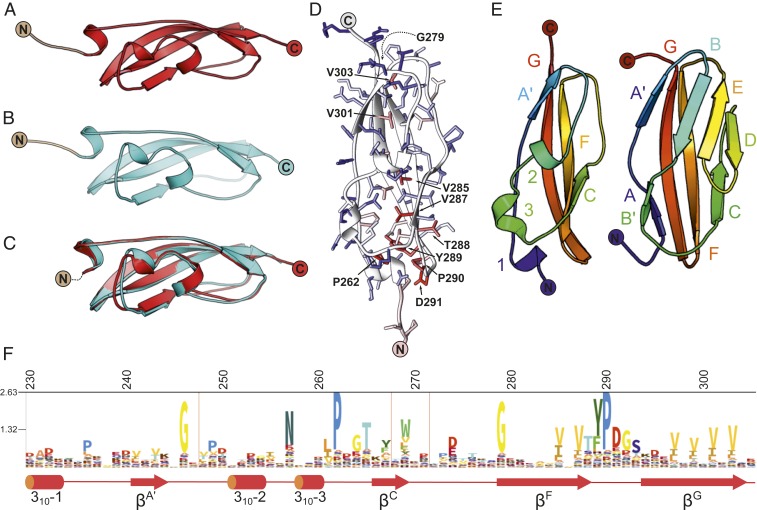
X-ray crystallography-determined structures of Rib domains. (*A*) RibR (red) and (*B*) R28N (cyan) (N-terminal nonnative tag stubs are shown in straw and gray). (*C*) Ca superposition of RibR and R28N. (*D*) RibR ribbon diagram, with sidechains rendered as sticks and colored by sequence conservation blue to red, where red is the most highly conserved. (*E*) A comparison of RibR (*Left*) with the He_PIG family fold (PDB ID code: 4nzj) (*Right*) illustrates the loss of 4 β-strands (4nzj B, B′, D, and E, using standard Ig strand nomenclature), replaced by a coil and 2 3_10_ helices. Ca rmsd 2.5 Å with 64 residues aligned. (*F*) An HMM sequence logo describing the Rib domain annotated with RibR residue numbers (above) and secondary structure below (3_10_ helices [cylinders] and β-strands [arrows]).

The structure of RibR was determined by experimental phasing and refined at 1.7 Å resolution (Table 1); the domain fold has a cuboidal structure consisting of a mixed 3_10_/β fold comprising a 4-stranded β-sheet curved around 3 short 3_10_-helices, with N and C termini pointing in opposite directions along the long axis of the anisometric fold (Fig. 2*A*). A 1.8-Å resolution structure of R28N (Fig. 2*B*) was solved using the structure of RibR as a search model; the Cα root-mean-square deviation (rmsd) between RibR and R28N is 1.0 Å (Fig. 2*C*).

**Table 1.** Data collection and processing statistics for Rib-SeMet, RibR, R28N, Rib2R, RibL, and RibS

	RibR SeMet	RibR	R28N	Rib2R	RibL	RibS
Data collection						
Diamond beamline/ wavelength, Å	I04/0.9794	I24/0.9778	I03/0.9202	I04/0.9795	I03/0.9763	I04/0.9795
Space group	P4_1_2_1_2	P4_1_2_1_2	P2_1_	P2_1_	C222_1_	P2_1_
Cell dimensions						
a, b, c, Å	a, b = 49.01, c = 144.50	a, b = 48.88, c = 144.57	a = 45.42, b = 43.56, c = 47.62	a = 37.83, b = 380.85, c = 37.85	a = 43.37, b = 147.81, c = 56.62	a = 30.1, b = 32.64, c = 62.81
α, β, γ, ^o^	α, β, γ = 90	α, β, γ *=* 90	α, γ = 90, β *=* 118.27	α, γ = 90, β = 91.6	α, β, γ = 90.00	α, γ = 90.00, β = 90.00
Resolution, Å	72.25–1.90 (1.94–1.90)	46.31–1.70 (1.73–1.70)	41.94–1.80 (1.86–1.80)	38.09–2.30 (2.38–2.30)	44.95–1.07 (1.09–1.07)	32.6–1.25 (1.27–1.25)
No. of unique reflections	14,731 (930)	20,225 (1,055)	15,119 (1,482)	47,115 (4,792)	78,337 (3,096)	17,716 (773)
Completeness, %	100 (100)	99.9 (100)	98.4 (97.8)	99.6 (99.6)	97.3 (77.9)	99.3 (90.4)
Multiplicity	8.3 (8.7)	6.2 (6.2)	4.0 (4.1)	6.9 (7.0)	6.9 (3.6)	6.7 (2.7)
*I*/σ(*I*)	8.1 (1.8)	6.3 (1.9)	8.0 (1.9)	8.3 (0.5)	13.2 (1.1)	19.2 (4.3)
*R*_merge_, %	21.2 (>100)	17.3 (>100)	12.9 (93.6)	16.3 (>100)	6.5 (>100)	4.6 (>100)
CC_1/2_	0.99 (0.74)	0.99 (0.60)	0.99 (0.64)	1.0 (0.43)	1.0 (0.31)	0.99 (0.96)
CC*	—	1.0 (0.87)	1.0 (0.88)	1.0 (0.77)	1.0 (0.77)	1 (0.99)
CC_anom_	0.822*	—	—	—	—	—
Refinement						
Resolution, Å		46.31–1.70	41.94–1.80	38.09–2.30	44.95–1.07	31.43–1.25
* R*_work_/*R*_free_, %		16.0/19.2	17.0/22.4	23.4/27.5	14.7/17.8	15.5/18.2
CC_work_/CC_free_		0.94/0.94	0.96/0.94	0.94/0.92	0.97/0.97	0.96/0.99
No. of reflections						
Total		20,156	15,115	47,105	78,305	17,665
Free		981	842	2,349	3,779	812
rmsd bond length, Å		0.009	0.007	0.009	0.006	0.0187
rmsd bond angles, °		1.06	0.85	1.09	0.88	2.076
No. of atoms						
Protein		2,370	1,258	9,391	3,051	1,128
Ligand		2	2	—	16	—
Solvent		291	295	32	381	75
Average B factors, Å^2^						
Protein		16.0	19.0	92.0	14.0	12.26
Ligand		14.5	25.7	—	45.4	—
Solvent		28.0	30.9	58.0	33.3	23.1
Ramachandran plot, %						
Preferred		100.0	100.0	98.4	99.5	98.7
Allowed		0.0	0.0	1.6	0.5	1.3
Outliers		0.0	0.0	0.0	0.0	0.0

Values in parentheses are for the outer shell. CC_1/2_, half dataset correlation coefficient; CC_anom_, CC between anomalous signals from half datasets; CC_work_, CC of the experimental intensities with the intensities calculated from the refined model; CC_free_, CC of experimental intensities of free reflections excluded from refinement with intensities calculated from the refined model. Empty cells and cells containing em dashes (—) infer that this value is not applicable to the dataset.

* Significant anomalous signal extends to a resolution of 3.13 Å (above CC_anom_ threshold 0.15).

### Conserved Sequence and Structural Features of the Rib Domain.

The Rib domain structure (Fig. 2*D*) and hidden Markov model (HMM) logo (18) reveal the location and identity of the conserved residues (Fig. 2*F*). A striking feature in the C-terminal part of the domain is 2 long antiparallel β-strands forming the central strands of the 4-stranded β-sheet. For example, 4 hydrophobic residues near the C terminus that are separated by 1 residue lock the C-terminal strand into the hydrophobic core of the domain. Similarly, 2 hydrophobic amino acids N-terminal to the conserved YP motif contribute to the hydrophobic core from the adjacent strand while the conserved proline in YP enables formation of the tight β-turn (*SI Appendix*, Fig. S1*A*).

### Rib Is an Example of Domain Atrophy.

The protein structure comparison service PDBeFold at the European Bioinformatics Institute (19) identifies the closest structural homolog of RibR as an immunoglobulin-like (Ig-like) fold from *Bacteroides thetaiotaomicron* SusCD complex protein BT2262 (PDB ID code: 5fq7 chain H, residues 9 to 88) having a Cα rmsd to RibR of 3.1 Å over 68 residues; other close structural homologs were also from the Ig-like superfamily. Comparison with the He_PIG family (PF05345) (Fig. 2*E*) shows the *Rib* domain fold is missing strands equivalent to the D and E strands characteristic of the B-D-E β-sheet of the typical Ig fold. The third strand (strand B) in the top sheet of 4nzj (He_PIG) is replaced by a coil and 2 3_10_ helices in the *Rib* structure. In common with He_PIG, strands A′, G, F, and C form the 4-stranded β-sheet. Thus, despite lacking 1 of the 2 β**-**sandwich sheets of the Ig fold, the Rib domain is clearly a member of the Ig-like fold superfamily, in agreement with the Pfam clan assignment (Pfam clan: CL0159). Given the narrow species distribution of the Rib family compared to the wide distribution of Ig-fold proteins, we suggest that *Rib* is an example of domain atrophy from an ancestral Ig fold, a rare phenomenon where core elements of secondary structure are lost (15).

### Ancestral Rib Domains.

**Fig. 3.**
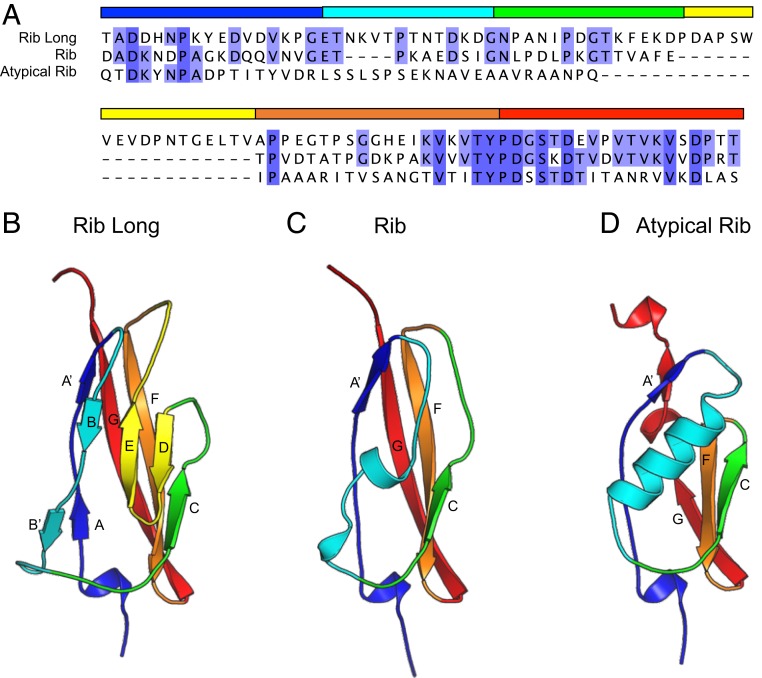
A comparison of the sequence (*A*) and topology of Rib Long (*B*), Rib (RibR) (*C*), and atypical Rib (aRib; SrpA) (*D*) structures. Structures were aligned and superposed using Matt (55). The structures are shown in the same orientation (N terminus down, C terminus up) and colored to illustrate homologous structural regions, and the sequence alignment is colored by amino acid identity. To highlight topological similarities and differences, the loop conformations of the domains have been smoothed.

We hypothesized that some members of the Rib domain that have long insertions in the position of the characteristic Rib D-E strand atrophy (Fig. 3*A*) may retain the full Ig fold and thus represent something akin to the ancestral form of the atrophied Rib domain. A surface protein from *L. acidophilus* (UniProt: Q5FIM8) contains both long (L) and standard (S) Rib domains (Fig. 1) with 45% sequence identity (*SI Appendix*, Figs. S1*B* and S2*A*). Thus, we solved the structure of “Rib Long” (RibL) (residues 1165 to 1268) and “Rib Standard” (RibS) (residues 1269 to 1329) at resolutions of 1.07 Å and 1.25 Å, respectively (Table 1). RibS belongs to the “Rib” subfamily, giving a Cα rmsd value of 0.6 Å for superposition with both RibR and RibR28N (*SI Appendix*, Fig. S2 *B* and *C*), versus a Cα rmsd of 2.3 Å for RibL (*SI Appendix*, Fig. S2*D*). As predicted, the Ig-fold D/E strands missing from RibR and R28N are also missing from RibS but are present in the RibL structure (*SI Appendix*, Fig. S2*D*). Thus, RibL comprises a full Ig-like fold which likely represents the ancestral state of the Rib domain, and RibS is an example of evolutionarily recent domain atrophy from RibL; this is remarkable because there are very few cases where we have the structure of both atrophied and nonatrophied domains that are closely related in sequence.

### Atypical Rib Domains.

Further sequence analyses identified the “unique subdomain” from *Streptococcus gordonii* serine-rich repeat protein GspB (20) as a distant relative of the Rib domain for which a structure has been previously determined (PDB ID code: 3qc6) with an rmsd of 2.95 Å to RibR. In addition, the SrpA adhesin from *Streptococcus sanguinis* contains a related domain with an rmsd of 2.4 Å to RibR (PDB ID code: 5eq2) (21). These structures define an atypical Rib (aRib) Pfam family: PF18938. The topology is similar to the Rib domain (Fig. 3 *C* and *D*); the antiparallel 3-stranded β-sheet at the C terminus of the domains is conserved, although smaller in aRib. A long α-helix replaces the short helical turn of the Rib domain, with an extended loop at the N-terminal end of the helix covering the missing space that results from the smaller β-sheet in aRib. The role of the aRib domain within the GspB and SrpA structures is uncertain; in SrpA, the domain is involved in dimerization, but the residues that mediate this interaction are not conserved in other Rib domains, suggesting this is not a shared feature (21). The topology of the F-G loop is similar in all 3 types of Rib domain (Fig. 3), and the discovery that the highly divergent aRib domain (from SrpA) shares a YPDGXXD motif with RibL and Rib (Fig. 3*A* and *SI Appendix*, Fig. S3) highlights the importance of these residues that appear to stabilize the β-turn and interaction between the central antiparallel strands.

The aRib domain (Pfam family: PF18938) is sometimes found tandemly arrayed and often associated with other known cell surface domains, such as DUF1542, FIVAR, Rib, and GA domains; 1,180 proteins are found in UniProtKB (release 2019_01) to contain the aRib domain, and it is found primarily in Firmicutes (86%) and Actinobacteria (12%), with some sporadic cases found in other bacterial groups. This compares to 2,405 proteins containing the Rib domain, with 77% being found in Firmicutes, and 19% in Actinobacteria and 2% in Proteobacteria.

**Fig. 4.**
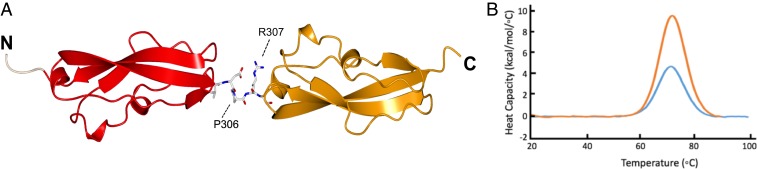
The interdomain linker in Rib2R is ordered and extended in the crystal lattice, maintaining an elongated structure with N and C termini at opposite ends of the molecule. (*A*) The structure of Rib2R (red, orange), with the linker region illustrated as cylinders, rendered by CCP4mg [atoms colored by type: C (white), N (blue), O (red)]. (*B*) Consistent with the limited interdomain interface observed in the crystal structure, the observed T_m_s are similar for the single (blue) (T_m_ 71.33 ± 0.05 °C) and 2-domain (yellow) (T_m_ 72.02 ± 0.09 °C) constructs; errors are estimated from fit to the data.

As Rib (RibR, R28N, and RibS), RibL, and aRib form 3 distinct Rib subfamilies (*SI Appendix*, Fig. S4), this begins to shed light on the evolutionary pathway of a fold where an ancestral Ig-like fold (RibL) has lost core secondary structures (RibR) and diversified in structure (aRib). The replacement of the typical B-D-E β-sheet of an Ig fold by an α-helix in the aRib domains, and a combination of loops and short helices in Rib, shows the remarkable structural malleability of the Rib family. The evolutionary mechanism termed domain atrophy has been shown to be extremely rare (15). In the atrophy of the Ig-like RibL, a significant loss of the structure (∼20% compared to RibR/RibS) is involved, but the domains remain thermostable with melting temperatures (T_m_s) of 78 °C and 88 °C for RibL and RibS, respectively (*SI Appendix*, Fig. S5), and 71 °C for RibR (Fig. 4*B*).

### Tandem Rib Domains Form a Relatively Rigid Rod.

Rib domains are often found tandemly arrayed (Fig. 1); thus, to examine the interdomain orientation, the structure of a 2-domain Rib construct (Rib2R) (residues 230 to 387) (Fig. 1) was solved (Fig. 4*A* and *SI Appendix*, Fig. S6*A*). The interdomain linker is short and consists of the residues DPRT (Fig. 4*B* and *SI Appendix*, Fig. S6*B*). The similarity of the T_m_ of RibR and Rib2R (Fig. 4*B*) is consistent with the lack of a large stabilizing interdomain interface.

To validate our RibR and Rib2R crystal structures, we conducted small angle X-ray scattering experiments with in-line size exclusion chromatography (SEC-SAXS) (*SI Appendix*, Fig. S7, *Insets*). Our analysis displays high quality model:data fits for both RibR and Rib2R (*SI Appendix*, Fig. S7 *A* and *B*), with χ^2^ values of 1.3 in each case. Analysis of the Guinier regions of both plots gives radius of gyration (*R*_*g*_) values of 14.7 Å and 26.5 Å, and distance distribution [*P*(*r*)] functions determine maximum particle dimensions (D_max_) of 57 Å and 97 Å for RibR and Rib2R, respectively. These calculated D_max_ values are consistent with our structural models (56 Å, RibR; 98 Å, Rib2R).

**Fig. 5.**
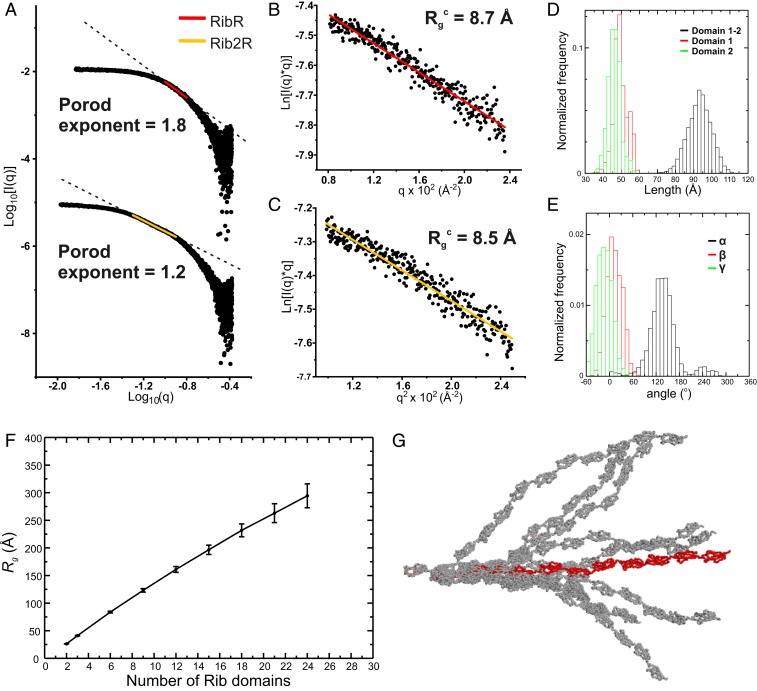
Rib2R has an elongated conformation in solution. (*A*) Porod plot for RibR (*Top*) and Rib2R (*Bottom*) from SAXS data. Porod exponents calculated from the negative slope of the linear region are displayed (RibR, red line, *R*^2^ = 0.98; Rib2R, yellow line, *R*^2^ = 0.99). (*B*) Modified RibR Guinier region, with cross-sectional *R*_g_ (*R*_g_^c^, annotated) calculated from fitted region (red line, *R*^2^ = 0.96; q*R_g_ range 0.8 to 1.3). (*C*) Modified Rib2R Guinier region, with *R*_g_^c^ calculated from fitted region (yellow line, *R*^2^ = 0.94; q*R_g_ range 0.8 to 1.3). (*D* and *E*) Molecular dynamics simulations of Rib2R. (*D*) Frequency of the distance between the 2 ends of the Rib2R tandem repeat and (*E*) frequency of the 3 angles identifying the rotation of domain 2 relative to domain 1 (see *SI Appendix*, Fig. S8*A* for definition of rotation angles). (*F*) A plot of increasing Rib domain number against *R*_g_ using simulated interdomain angles shows a linear trend consistent with elongation. (*G*) Ensemble of structures of a 12-domain Rib construct obtained by replicating domain 1 using translations and rotations observed during the MD simulation of Rib2R.

To assess the solution conformation of the Rib fold, we calculated both Porod exponents and cross-sectional *R*_g_ values (*R*_g_^*C*^) for RibR and Rib2R from our SAXS data (Fig. 5*A*). RibR displays a Porod exponent of 1.8, consistent with a rod-like particle tending toward a “flat disk”-like shape. Rib2R exhibits a Porod exponent of 1.2, consistent with scattering by a rod-like particle (22, 23). Importantly, both particles display very similar *R*_g_^C^ values (8.7 Å, RibR; 8.5 Å, Rib2R) (Fig. 5 *B* and *C*), suggesting that Rib2R forms an elongated molecule represented by 2 RibR molecules stacked end-on-end.

To further assess the interdomain orientation, we performed molecular dynamics (MD) simulations of fully solvated Rib2R at room temperature (303 K) (Fig. 5 *D* and *E*). During a 200-ns simulation, the 2-domain structure remains stable. The rmsd from the crystal structure fluctuates by up to 10 Å while that of the individual domains varies by ∼2 Å, showing that the mutual arrangement of the domains fluctuates in time around the crystal structure. Remarkably, the 2-domain structure remains extended on average, with an end-to-end distance of 96.2 ± 9.5 Å (Fig. 5*D*), which is consistent with both the D_max_ calculated from SAXS (97 Å) and the length of the crystal structure (98 Å). Modeling of the 3 interdomain rotation angles (*SI Appendix*, Fig. S8*A*) suggests that, while a wide range of long axis (*x*) rotations (α) are accommodated, rotations around the *y* and *z* axes (β, γ) occupy a more narrow distribution (Fig. 5*E* and *SI Appendix*, Fig. S8 *B* and *C*). MD model-to-SAXS data fits are consistent with an extended Rib2R solution conformation, with low χ^2^ values obtained for a wide range of α rotations (*SI Appendix*, Fig. S8*B*) but for only a narrow distribution of β and γ angles (*SI Appendix*, Fig. S8*C*). Models of multidomain Rib constructs using the MD-obtained range for α, β, and γ show an approximately linear dependence of *R*_g_ on Rib domain number (Fig. 5*F*) and elongated structures (Fig. 5*G*). Taken together, our data support the presence of an elongated rod-like structure for Rib that projects a functional N-terminal domain away from the cell surface at a variable distance dependent on the number of Rib domains present.

Given the lack of interdomain stabilization in Rib2R, rigidity in the interdomain linker is likely to be influential in maintaining the interdomain interface. In alpha C (UniProt: Q02192), the linker residues are predicted to be PKPVP; the presence of 3 proline residues is likely to result in an even higher degree of restricted interdomain dynamics. It is interesting to consider how representative these linkers are of the Rib domain proteins in general. The most common linker length between tandem Rib domains is shorter at just 2 residues (*SI Appendix*, Fig. S9), which suggests that most tandem Rib domains will be more constrained than in Rib2R and thus are likely to also form relatively rigid, extended arrays.

In summary, the detection of structural domains within highly repetitive protein sequences is challenging. The Rib (RibR) structure revealed that the original Pfam analysis successfully predicted the C-terminal residues of the domain, but the N terminus of the HMM needed to be extended by ∼11 amino acids. This has been adjusted within the Pfam database, which now also includes new families representing the RibL and aRib domain. These changes, motivated by our structural analysis, will enhance the accuracy and completeness of annotation of Rib domains across known protein sequences.

Rib domains are commonly found tandemly arrayed in surface-attached proteins of gram-positive bacteria and have been suggested to adopt an Ig-like fold (24, 25); Rib domain-containing alpha-like family proteins and domain number variability have been linked to infection and immune evasion. Here, bioinformatic and structural analyses of single Rib domains reveal a fold that is a significantly atrophied version of the Ig fold. A longer Rib domain contains the full Ig fold and likely represents an ancestral preatrophy structure, thus providing a rare example of a closely related pair of structures of an atrophied domain with its ancestral form. The Ig domain is one of the most recognized protein folds and is known to undergo a variety of elaborations with additional β-strands and loops. Prior cases where domain atrophy of Ig domains is suggested by structure analysis have been noted (26). However, the loss of core secondary structures that can be convincingly linked by sequence to the complete Ig fold has not been previously observed. In tandem, Rib domains assemble head-to-tail and with limited interdomain flexibility (Fig. 5). Combined with domain number variation, this suggests that arrayed domains in the infection-linked alpha-like proteins of GBS (5) will form an elongated rod that projects the N-terminal cell binding domain (Figs. 1 and 5*F*) to variable distances from the bacterial surface. Such differential projection might be linked to the previously suggested immune evasion employed by GBS (12) through changes in protein accessibility or could also play a steric role by projecting beyond and thus regulating accessibility to proteins closer to the bacterial cell surface, as has been suggested for elongated cell surface proteins of *Staphylococcus aureus* (27, 28).

## Materials and Methods

### Cloning.

A clone of *S. agalactiae* Rib was kindly provided by Prof. Gunnar Lindahl, Division of Medical Microbiology, Lund University, Lund, Sweden (GenBank: U58333) coding for 2 repeats from the Rib protein (UniProt: P72362; amino acids 230 to 387; Rib2R). For this work, the coding sequence for a single Rib repeat (amino acids 230 to 308; RibR) was amplified by PCR using primers (*SI Appendix*, Table S1). The product was subcloned into the pGEX-6P-1 vector (GE Healthcare) by restriction endonuclease digestion with enzymes NdeI and XhoI followed by ligation-dependent cloning, generating an in-frame fusion with an N-terminal glutathione *S*-transferase (GST) tag and human rhinovirus 3C protease specific linker (GST-3C-RibR). Owing to difficulties separating the GST tag from Rib2R during protein purification, Rib2R was amplified using primers (*SI Appendix*, Table S1) and incorporated downstream of a Hexa-His and 3C protease-specific linker sequence (MGSSHHHHHHSSGLEVLFQGPAM) in the pETYSBLIC3c vector backbone (29) by In-fusion cloning (Clontech) as per the manufacturer’s instructions. For crystallization, a mutation in the affinity tag linker of Rib2R was also prepared using primers (*SI Appendix*, Table S1) to generate the sequence MGSSHHHHHHSSGLEVLFQGPLGS. The *Escherichia coli* optimized coding sequence for *S. pyogenes* R28 protein (UniProt: Q9XDB6) Rib domain (amino acids 230 to 307; R28N) was synthesized (Integrated DNA technologies) and PCR amplified using primers (*SI Appendix*, Table S1) for cloning downstream of a His-tag and tobacco etch virus (TEV) protease-specific linker sequence (MGSSHHHHHHSSSENLYFQS) in the pET28a vector backbone by In-fusion cloning. The *E. coli* optimized coding sequences for RibL (UniProt: Q5FIM8 1165 to 1268) and RibS (residues 1269 to 1329) were synthesized (Genewiz) and inserted into the pETYSBLIC3c vector backbone (29) using primers (*SI Appendix*, Table S1) and the In-Fusion cloning method, as described for Rib2R (see above).

### Protein Expression and Purification.

GST-3C-RibR was transformed into BL21(DE3) cells, grown in lysogeny broth (LB) supplemented with ampicillin (100 μg/mL) to OD_600_ 0.6 and expression induced by addition of 0.1 mM isopropyl β-d-1-thiogalactopyranoside incubated for 18 h at 30 °C with shaking. SeMet labeling was performed as described in ref. 30 with protein expression induced in *E. coli* B834 (DE3) cells in minimal media supplemented with 40 μg⋅mL^−1^ l-selenomethionine and purification performed as per the native protein. Cells were harvested by centrifugation at 4,500 × *g*, resuspended in phosphate-buffered saline (PBS), and lysed by sonication and lysate clarified by centrifugation at 48,000 × *g* 30 min at 4 °C. A GST-Trap4b column (GE Healthcare) was equilibrated in PBS, loaded with lysate at a flow rate of 1 mL⋅min^−1^, and washed with 50 mL of PBS. Bound protein was eluted in PBS supplemented with 10 mM reduced glutathione and was dialyzed against PBS for 18 h at 4 °C following the addition of 3C protease to remove the tags (1:100 wt/wt 3C:RibR). Cleaved RibR was concentrated by centrifugal filtration in a Vivaspin 3-kDa molecular weight cut-off (MWCO) filter (Sartorius) and separated from GST and 3C protease by size exclusion chromatography (SEC) using a Superdex 75 16/60 column (GE Healthcare) equilibrated in 20 mM sodium potassium phosphate buffer, pH 7.5. Elution of RibR was monitored by absorbance at 220 nm. RibR was subsequently dialyzed into 20 mM Tris, 50 mM NaCl at pH 7.5, and concentrated by centrifugal filtration with a Vivaspin 3-kDa MWCO PES membrane (Sartorius) for crystallization screening.

pETYSBLIC3c-6His-3C-Rib2R, pETYSBLIC3c-6His-3C-RibL, or pETYSBLIC3c-6His-3C-RibS were transformed into BL21(DE3) cells and grown in LB supplemented with kanamycin (50 μg⋅mL^−1^). Induction of protein expression, cell harvesting, and cell lysis were performed as described above. 6His-3C-Rib2R, 6His-3C-RibL, and 6His-3C-RibS were purified by standard immobilized metal affinity chromatography (IMAC) methods with gradient elution. The affinity tag was removed by application of 3C protease (1:100 wt/wt), and the sample was purified to homogeneity by SEC in buffer comprising 20 mM Tris, pH 8, 150 mM NaCl. pET28-His-TEV-R28N was transformed into BL21(DE3) cells, and protein expression, cell lysis, and purification were performed as described for Rib2R, with application of TEV (1:100 wt/wt) for removal of the poly-His tag and SEC buffer comprising 20 mM Tris, pH 7.5, 150 mM NaCl.

### Protein Crystallization.

RibR, RibR-SeMet, R28N, Rib2R, RibL, and RibS were crystallized by sitting-drop vapor diffusion at concentrations of ∼30 mg⋅mL^−1^, ∼65 mg⋅mL^−1^ (Rib2R), and 34 mg⋅mL^−1^ (RibL and RibS); 150 nL each of protein and reservoir solution were mixed using a Mosquito robot (TTP Labtech) into Swissci MRC 96-well trays, alongside 54 μL of reservoir solution. Crystallization trays were incubated at 291 K (RibR and RibR-SeMet) and 279 K (R28N, Rib2R, RibL). Crystals of RibR and SeMet RibR were obtained in the Index screen (Hampton Research) in 0.1 M citric acid, pH 3.5, and 2 M ammonium sulfate (RibR) and 3.5 M sodium formate, pH 7.0 (SeMet RibR). R28N crystallized in the JCSG+ screen (31) in 0.15 M potassium bromide and 30% polyethylene glycol (PEG) 2000 MME, and crystals of Rib2R grew in 3 M ammonium sulfate. RibL was crystallized in 0.2 M ammonium sulfate, 30% PEG 4000 at a concentration of 34 mg⋅mL^−1^. RibS was crystallized in 2 M ammonium sulfate, 2 M NaCl at a concentration of 34 mg⋅mL^−1^. RibR and Rib2R protein crystals were cryoprotected in 4 M sodium malonate, RibL in reservoir solution supplemented with 20% glycerol, and R28N in reservoir solution supplemented with 10% ethylene glycol, prior to vitrification in liquid nitrogen for data collection. RibR-SeMet was vitrified without cryoprotectant.

### Structure Determination.

Data collection was performed at the Diamond Light Source (Oxfordshire, United Kingdom), beamlines as shown in Table 1. Data integration, scaling, and reduction were performed using the Xia2 pipeline (32–36). The structure of RibR was solved by SeMet Single-wavelength Anomalous Dispersion phasing. Substructure solution, phasing, and model building were performed utilizing Crank2 as implemented in CCP4i2 (37, 38) and autobuilt by ARP/wARP (39). This model was refined against higher resolution native data for RibR using REFMAC5 (40). Crystal structures of R28N and Rib2R were solved by molecular replacement (MR) in MOLREP (41) using the refined model of RibR. Initial models were built and refined using BUCCANEER (42) and REFMAC5 (40), respectively. PROSMART was utilized to generate geometric reference restraints based on RibR for the refinement of Rib2R (43). RibR and R28N were refined to completion in PHENIX (44) and Rib2R using autoBUSTER (45).

For the solution of RibL, a conserved fragment of RibR corresponding to residues 60 to 71 was selected as a molecular replacement model for PHASER (46) and gave borderline scores (TFZ = 6, LLG = 64). This fragment was used as an input model for the program FRAGON (47) to solve the structures of both RibL and RibS, resulting in density-improved phases and maps that could be autotraced using ARP/wARP (39). A sequence-updated version of the RibL model was then built into the refined map using BUCCANEER (42). The RibL model was refined with 30 cycles of jelly body refinement in REFMAC5 (40) and was subsequently refined to completion in Phenix (44). RibS was refined to completion with REFMAC5 (40). All structures were manually built between refinement cycles using COOT (48), and stereochemistry was validated using MOLPROBITY (49). Structure superposition was performed using Gesamt (50) executed in ccp4i (51). Structural figures in this paper were generated using CHIMERA (52), Pymol (Schrödinger LLC, Version 2.1.0), and CCP4mg (53). Refinement statistics are reported in Table 1.

### Determination of T_m_.

For RibR and Rib2R, protein samples (1 mg/mL in 20 mM NaH_2_PO_4_, pH 7.4) and well-matched buffer were degassed for 10 min at 10 °C using a Thermovac degasser (MicroCal). Differential scanning calorimetry (DSC) scans were acquired on a MicroCal VP-DSC calorimeter (Malvern Panalytical) with a scan rate of 90 °C/h. The reversibility of thermal unfolding was verified by repetitive scans on the same sample. A buffer–buffer scan was subtracted from each protein scan, followed by normalization for protein concentration and subtraction of a progress baseline for sample baseline correction. Melting temperatures (T_m_s) were derived by fitting the unfolding endotherm using a 2-state unfolding model for data above 20 °C. Data analysis was performed using MicroCal Origin 5.0.

The T_m_ values for RibL and RibS were determined using a Prometheus (differential scanning fluorimetry [DSF]) instrument (NanoTemper) at a protein concentration of 1 mg/mL in 20 mM Tris, 150 mM NaCl, pH 7.5, using a sample volume of 10 µL. Samples were heated to 95 °C at a rate of 1 °C/min to generate an unfolding profile.

### Molecular Dynamic Simulations.

Molecular dynamic (MD) simulations were performed starting from Rib2R chains A and C (PDB ID code: 6S5Y). Simulations were performed using the CHARMM36m (54) force field and NAMD (55). The protein was energy minimized and solvated in a periodic orthogonal box 134 Å × 58 Å × 48 Å, needed to guarantee a layer of at least 12 Å solvent around the elongated protein. After a 1-ns equilibration, the systems were simulated at 303 K for 200 ns. Simulations were performed in the isothermal–isobaric ensemble where the temperature was kept constant on average through a Langevin thermostat, and the pressure was set to 1 atm through an isotropic Langevin piston manostat. Rotation angles α, β, and γ were calculated by decomposing the 4D transformation matrix obtained from aligning the 2 Rib domains within PyMOL. Multidomain Rib models were constructed by applying the inverse transformation matrices to each Rib monomer added to the growing chain.

### Small Angle X-Ray Scattering.

SAXS experiments were performed at B21, Diamond Light Source, United Kingdom, over a momentum transfer range (*q*) of 0.01 Å^−1^ < *q* < 0.4 Å^−1^. Scattering intensity (*I* vs. *q*, where *q* = 4πsinθ/λ and 2θ is the scattering angle) was collected using a Pilatus 2M detector, with an incident beam energy of 12.4 keV and a beam-to-detector distance of 4,014 mm. RibR and Rib2R samples were injected onto an in-line Shodex KW402 column equilibrated in 10 mM Hepes, 150 mM NaCl, 3 mM KNO_3_, pH 7.5, both at concentrations of 10 mg⋅mL^−1^. Data processing and reduction and calculation of *R*_g_^c^ plots [Ln(I*q) vs. *q*^2^] were performed using the program Scatter (https://github.com/rambor/scatter3.git). *R*_g_ {Ln[*I*(*q*)] vs. *q*^2^} and distance distribution [*P*(*r*) vs. *r*] plots were calculated with Primus (56). Tag residues unresolved in the crystal structure of Rib2R were added via Modeler (57), and all-atom ensembles were generated with Allosmod (58). For each example, 50 independent pools of 100 models were created, and calculating and fitting of theoretical scattering curves from models to experimental data were performed using FoXS (59). This process was automated using Allosmod-FoXS. Estimation of the useful angular range of the data was calculated using the program Shanum (60). Plots were generated using OriginPro v9.5.5.409 (OriginLab), as were the gradients of the linear regions of double logarithmic plots.

### Data Availability.

Structures were deposited in the Protein Data Bank, https://www.wwpdb.org/ (PDB ID codes 6S5X [RibR], 6S5Y [Rib2R], 6S5Z [R28N], 6S5W [RibL], and 6SX1 [RibS]). Other data presented are available from the corresponding authors on reasonable request.

## Supplementary Material

Supplementary File
