# Supplementary material for: Defining the remarkable structural malleability of a bacterial surface protein Rib domain implicated in infection
Source: Proc Natl Acad Sci U S A. 2019 Dec 9;116(52):26540–8. doi: 10.1073/pnas.1911776116 (PMC6936399; doi:10.1073/pnas.1911776116)
Supplement: Supplementary File [file pnas.1911776116.sapp.pdf]

PNAS
www.pnas.org

Supplementary Information for 'The Rib domain: Defining the remarkable structural malleability of a bacterial surface protein domain implicated in infection.'

Fiona Whelan, Aleix Lafita, Samuel C. Griffiths, Rachael E. M. Cooper, Jean L. Whittingham, Johan P. Turkenburg, Iain W. Manfield, Alexander N. St John, Emanuele Paci, Alex Bateman, and Jennifer R. Potts

Jennifer R. Potts
Email: jennifer.potts@york.ac.uk

This PDF file includes:

Figures S1 to S9
Table S1

www.pnas.org/cgi/doi/10.1073/pnas.1911776116

Table S1

Construct	Forward Primer	Reverse Primer
RibR	5′ TACGCACATATGGATG CCGATAAGAATGATCCAGCAGG 3′	5′ TTTAGTCTCGAGCTA TGTACGCGGATCGACAACC 3′
Rib2R	5′ TCCAGGGACCAGCAATGGATG CCGATAAGAATGATCCAGCAGG 3′	5′ TGAGGAGAAGGCGCGTCAT GTACGCGGATCGACAACC 3′
Rib2R (crys)	5′ CTGTTCCAGGGACCACTGGCAGCG ATGCCGATAAGAATGATCCAGCAGG 3′	5′ TGAGGAGAAGGCGCGTCAC GGGAATTCTTATTATGTACG 3′
R28N	5′ TCCAGAGCGGAGCAATGGATAAAAT CAAATACTCACCGGAAGC 3′	5′ TGAGGAGAAGGCGCGTTA GGTTTTCTTGCTGGTGACGG 3′
RibL	5’ TCCAGGGACCAGCAATGGGCCAGA CCGCGGATGATC 3’	5’ TGAGGAGAAGGCGCGTTAC GGGGTGGTCGGATCGCTC 3’
RibS	5’TCCAGGGACCAGCAATGGGCCAGAC CGATGCGGATAAATATAC 3’	5’TGAGGAGAAGGCGCGTTACG GGGTGGTCGGATCGGTC 3’

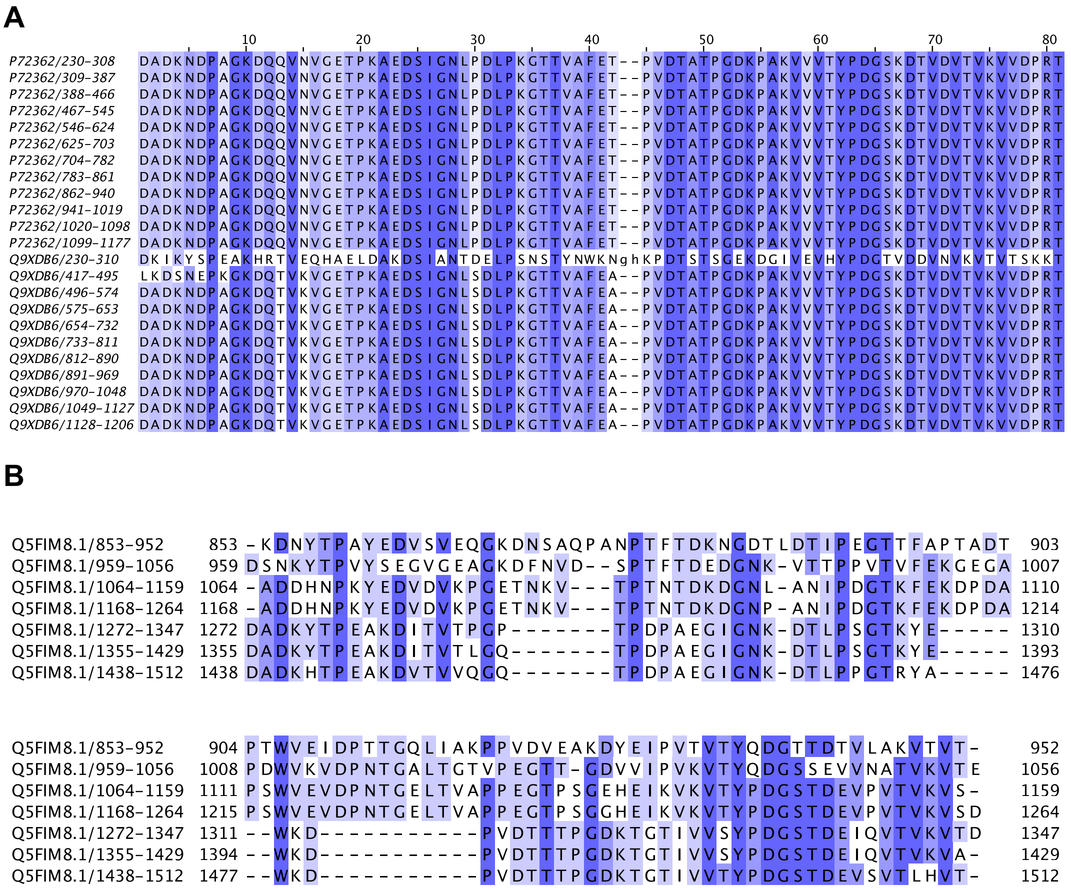

Fig. S1. Multiple sequence alignment of the Rib domains from the Rib and R28 proteins (*A*) and from the surface protein from *L. acidophilus* (UniProt: Q5FIM8, *B*). The alignment was created with HMMER and has been visualised using the Jalview software with percentage identity colouring.

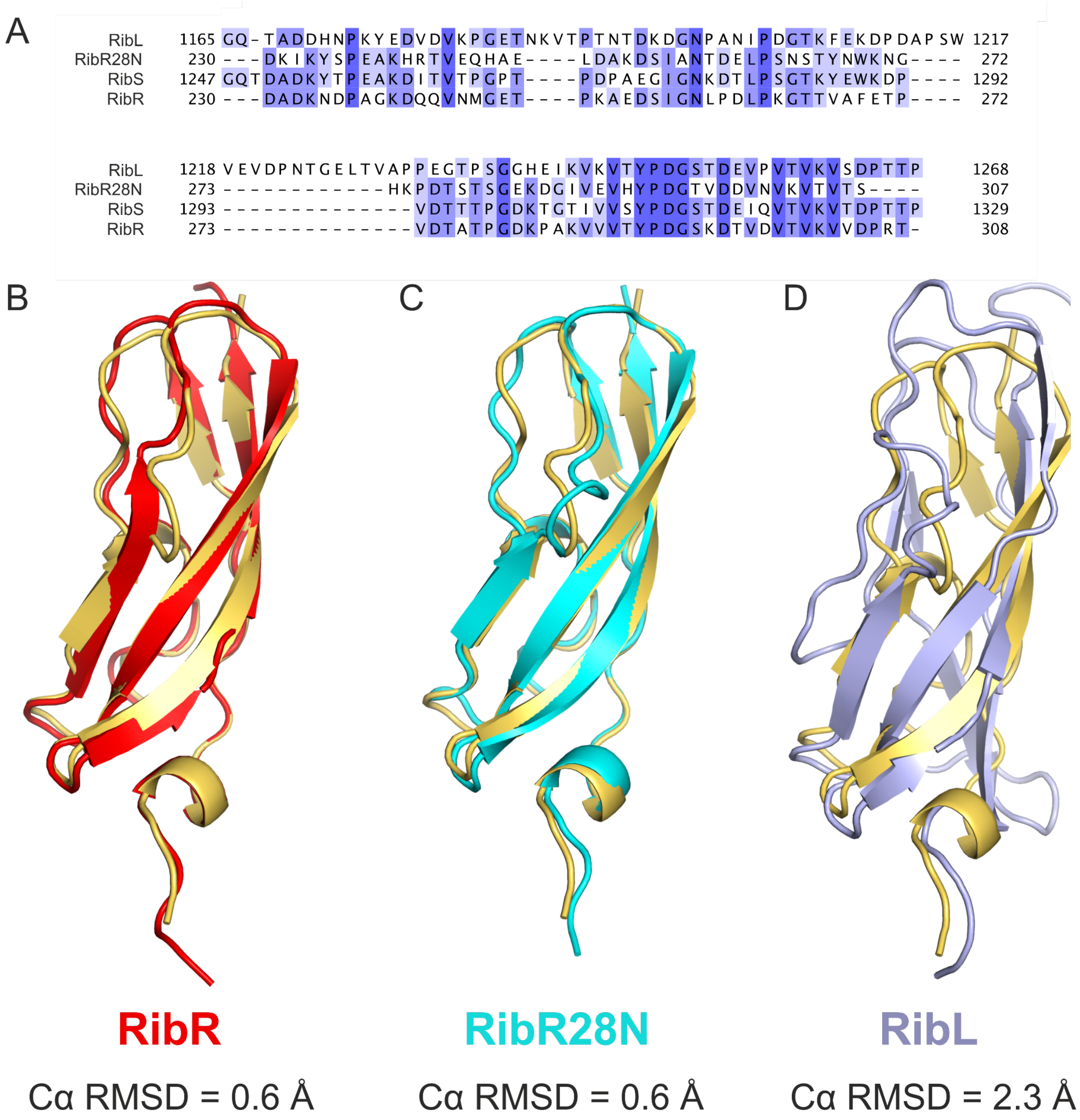

Figure S2. Structural superposition of Rib domain structures. (*A*) Sequence alignment of Rib structures solved in this study. Conserved residues are boxed and the YPDGxxD motif is highlighted in yellow. (*B-D*) Structural superposition of RibR (*B*; red, 70 Cα atoms), RibR28N (C; cyan, 69 Cα atoms) and RibL (*D*; violet, 53 Cα atoms) with RibS (yellow). Sequence alignments were generated using Clustal Omega and structural superpositions calculated using Pymol (Version 2.1.0).

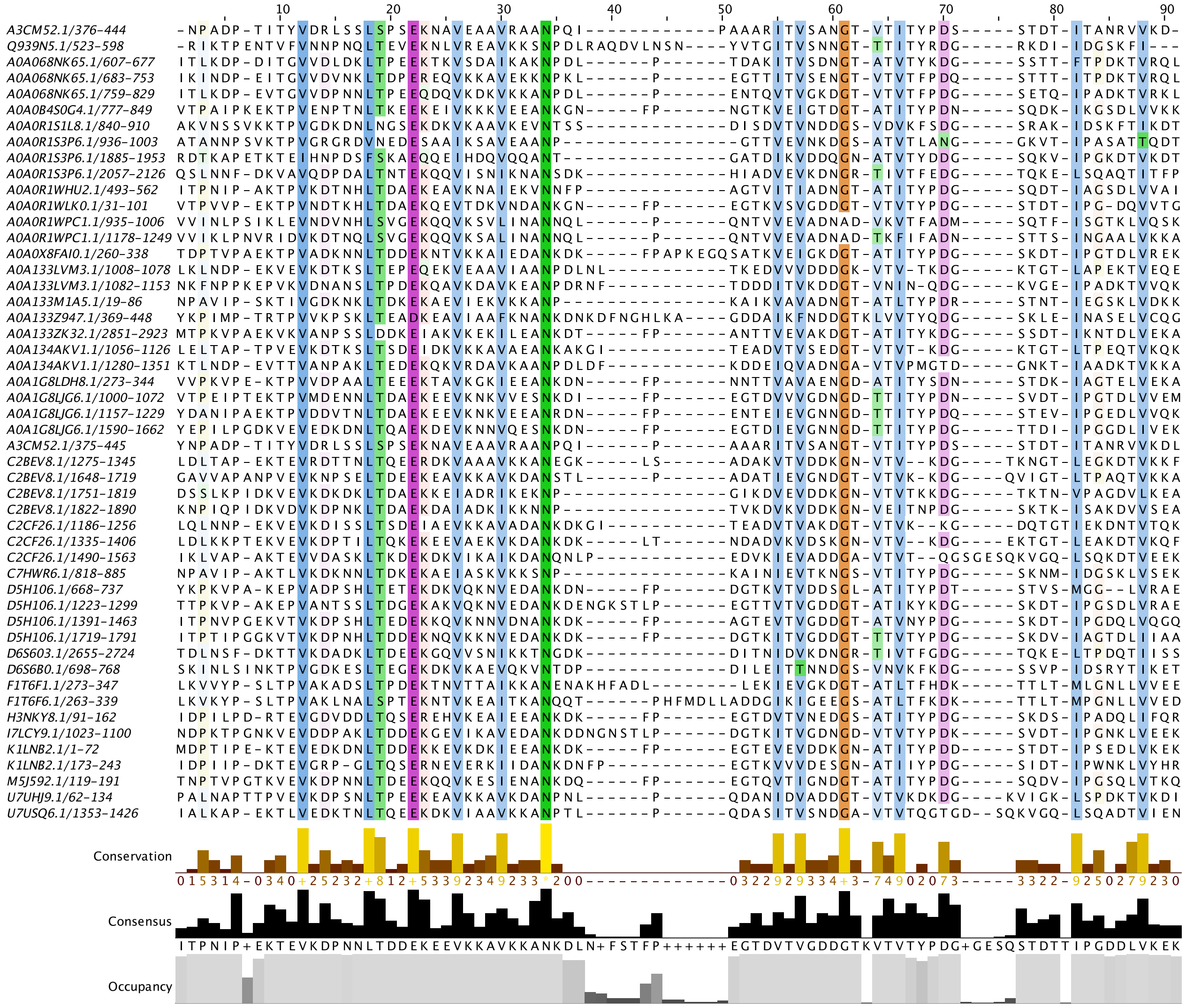

Fig. S3. Multiple sequence alignment taken from the Pfam seed alignment for the aRib domain. The alignment has been visualised using the Jalview software with Clustal colouring. Sequence identifiers are UniProt accession numbers followed by the start and end residue of the domain sequence. The level of sequence conservation, the consensus sequence and occupancy of each alignment column are shown in the tracks at the bottom of the alignment.

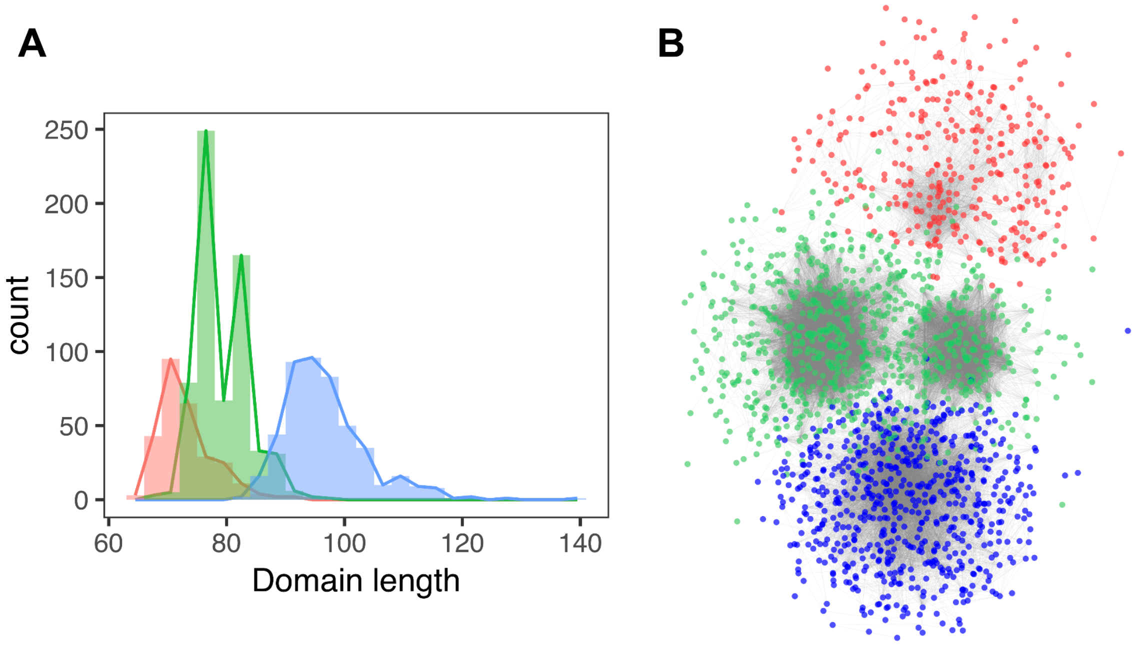

Fig. S4. Comparison of the three Rib domain families: Rib Long (blue), Rib (green) and atypical Rib (red). *(A)* Lengths of UniProt hits show overlapping but distinct distributions, consistent with the experimental structures. *(B)* Domain sequence similarity network from BLAST all-against-all analysis. Nodes are domain sequences coloured by Rib family and edges are significant BLAST hits. Network connectivity analysis shows clustering of domains within each family and that atypical Rib domains are more closely related to Rib than to RibL.

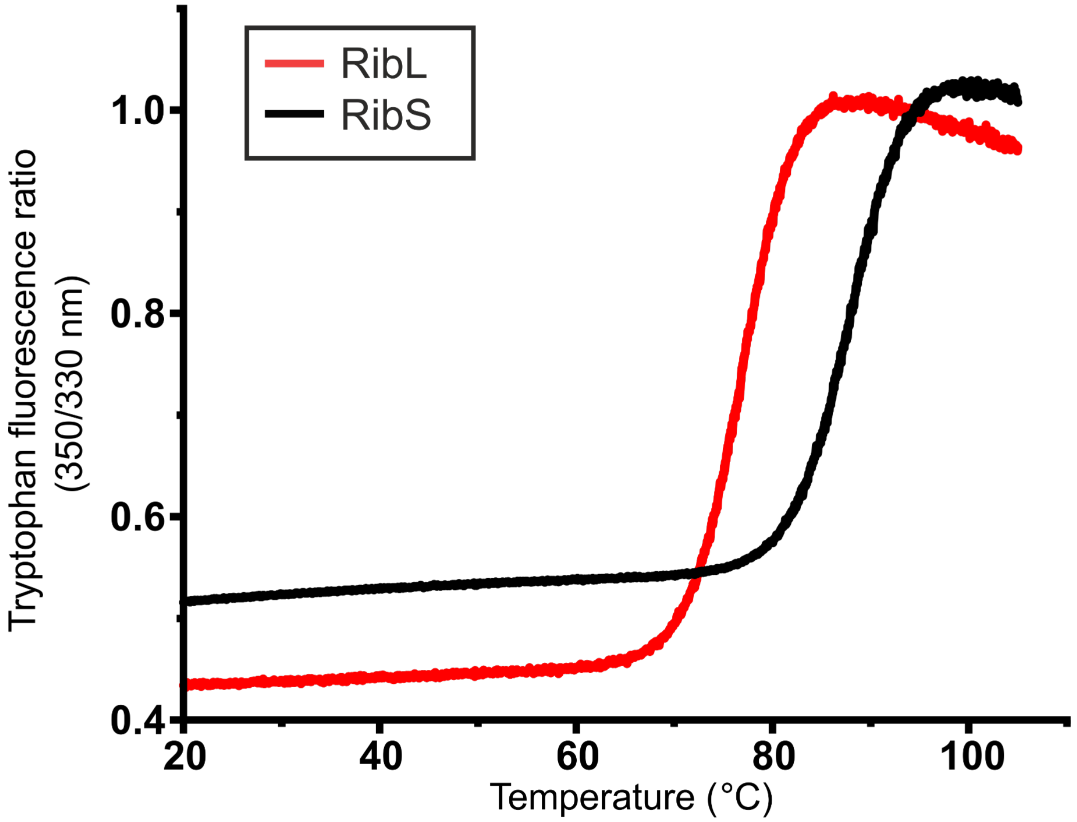

Fig. S5. Thermal denaturation experiments of RibL (red) and RibS (black) using nanoDSF. Melting temperature (T_m) values – RibL = 78 ± 0.1°C; RibS = 88 ±0.1°C; error is based on the average of two replicates.

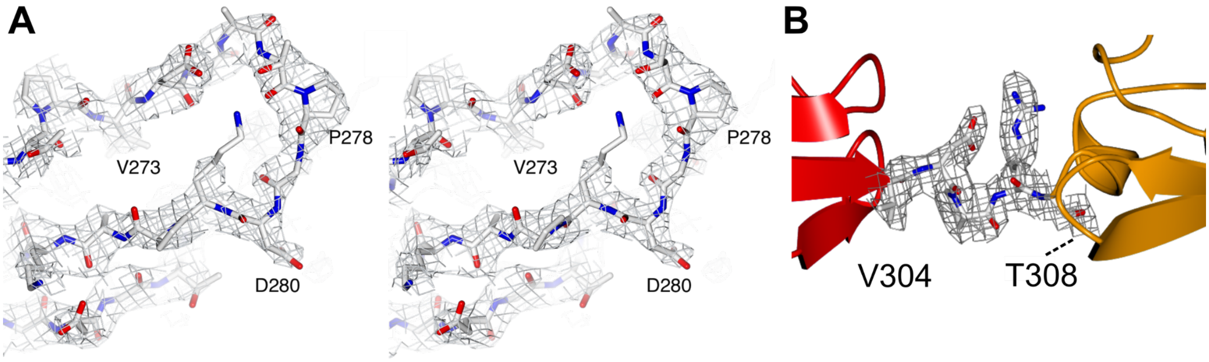

Fig. S6 X-ray crystal structure electron density of Rib2R. (*A*) Stereo image of the refined electron density and (*B*) the refined interdomain linker density V304—T308 (Chain B, $2F_o$-F_c, 1.0σ, grey). Both figures were rendered by CCP4mg.

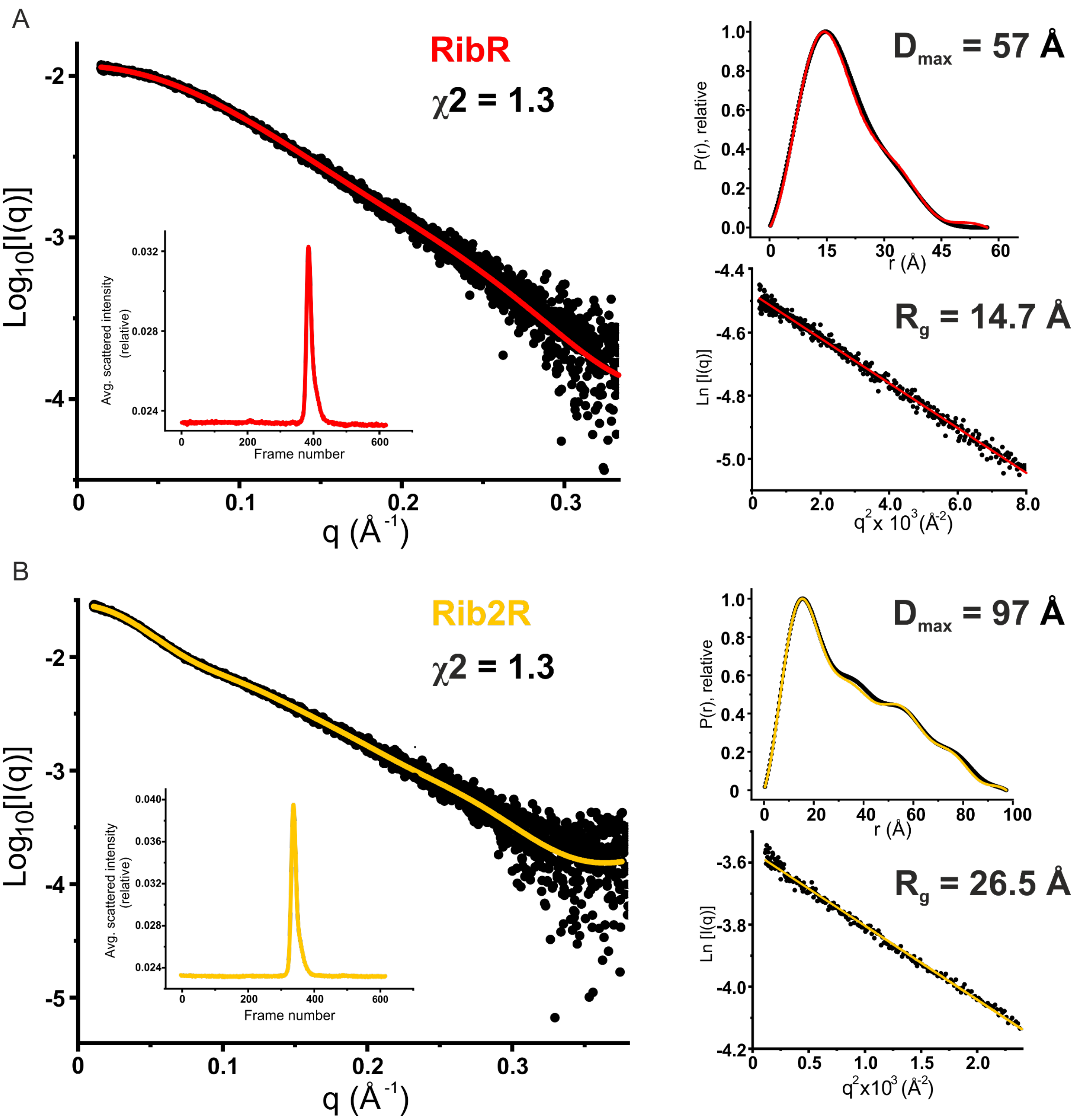

Fig. S7. Validation of RibR and Rib2R crystal structures via SAXS. (*A*) (left) Fit of the RibR model to experimental scattering data. Experimental (black) and calculated (red) scattering curves are displayed to a maximal momentum transfer of q = 0.335 $Å^{-1}$, with fit value ($χ^2$) annotated. The data are accounted for by a single model of RibR. A SEC trace of RibR sample injection is displayed (inset). Normalised pair-distance distribution function (experimental – black, model – yellow), with the calculated maximum intra-particle diameter (D_{max}) displayed (real space R_g = 15.0±0.5 Å, *I*(0) = 0.012) (top right). The linearity of the Guinier region confirms monodispersity (q^*R_g range 0.2-1.3; R_g = 14.7 Å, *I*(0) = 0.011) (bottom right). (*B*) (left) Fit of the Rib2R model to experimental scattering data. Experimental (black) and calculated (yellow) scattering curves are displayed to a maximal momentum transfer of q = 0.376 $Å^{-1}$, with fit value ($χ^2$) annotated. The data are accounted for by a single model of Rib2R, modelled using Allosmod-FoXS to add missing residues and calculate model:data fit values. A SEC

trace of Rib2R sample injection is shown (inset). Normalised pair-distance distribution function ($P(r)$) (experimental – black, Allosmod-FoXS model – yellow), with the calculated maximum particle dimension (D_{max}) displayed (real space R_g = 28.3±0.7 Å, $I(0)$ = 0.029) (top right). The Guinier region (inset) confirms monodispersity ($q*R_g$ range 0.3-1.3; R_g = 26.5 Å, $I(0)$ = 0.028) (bottom right).

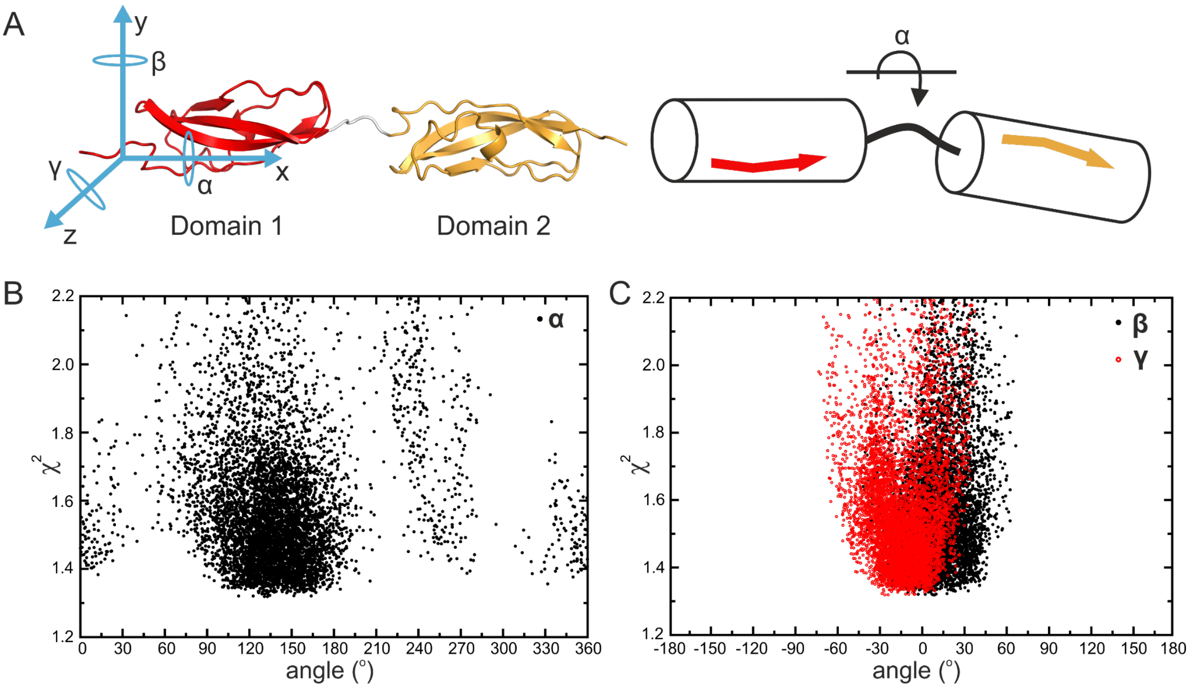

Fig. S8. Domain-domain rotation of Rib2R. (*A*) Definition of three domain-domain rotation angles α, β and γ along the x-, y- and z-axes respectively (left). For example, domains can rotate about α, displayed using cylinders (right). Red and yellow arrows signify the first strand of the long β-hairpin at the C-terminus of each Rib domain respectively. (*B*) Fit of simulated Rib2R models with variation of angle α to SAXS data. (*C*) Fit to SAXS data of snapshot from the MD simulation of Rib2R as a function of the angle α (*B*) and of angles β and γ (*C*).

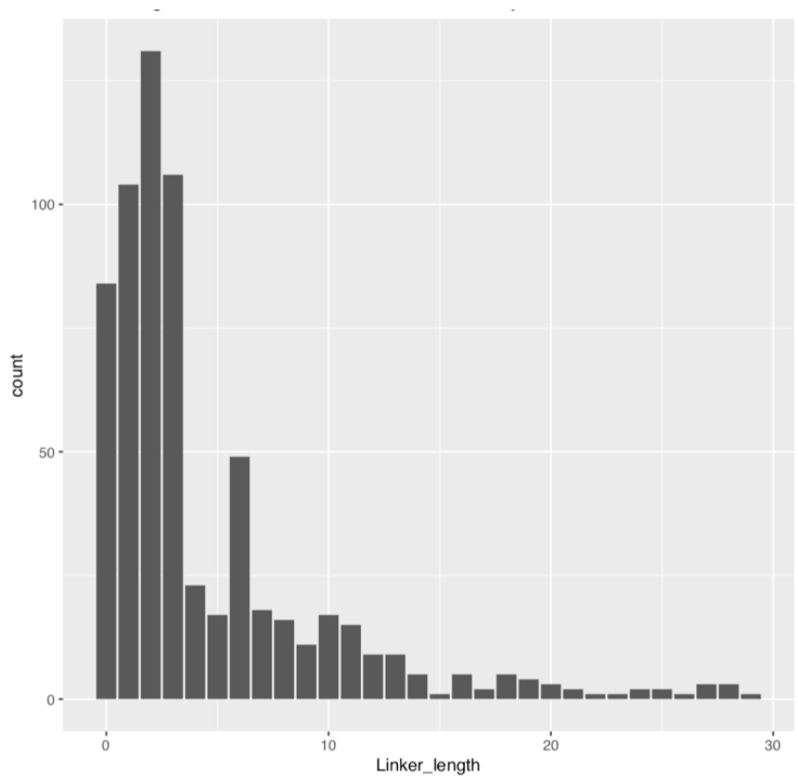

Fig. S9. The distribution of linker lengths between tandem Rib domains. The X axis shows the number of residues between the predicted end of one domain and the start of the next. The Y axis shows the frequency of linkers for each length.
